# Confronting the human papillomavirus–HIV intersection: Cervical cytology implications for Kenyan women living with HIV

**DOI:** 10.4102/sajhivmed.v24i1.1508

**Published:** 2023-10-27

**Authors:** James M. Kangethe, Stephen Gichuhi, Eddy Odari, Jillian Pintye, Kenneth Mutai, Leila Abdullahi, Alex Maiyo, Marianne W. Mureithi

**Affiliations:** 1 Consortium for Advanced Research Training in Africa (CARTA), Nairobi, Kenya; 2 Department of Medical Microbiology and Immunology, Faculty of Health Sciences, University of Nairobi, Kenya; 3 Comprehensive Care Center for HIV, Kenyatta National Hospital, Nairobi, Kenya; 4 Department of Ophthalmology, Faculty of Health Sciences, University of Nairobi, Nairobi, Kenya; 5 Department of Medical Microbiology, Faculty of Health Sciences, Jomo Kenyatta University of Agriculture and Technology, Nairobi, Kenya; 6 Department of Biobehavioral Nursing and Health Informatics, University of Washington, Seattle, United States of America; 7 Research and Policy Development, African Institute for Development Policy, Nairobi, Kenya; 8 Center for Virus Research, Kenya Medical Research Institute, Nairobi, Kenya; 9 KAVI Institute of Clinical Research, Faculty of Health Sciences, University of Nairobi, Nairobi, Kenya

**Keywords:** high-risk human papillomavirus, cervical cytology, cervical cancer, women living with HIV, antiretroviral therapy, Kenya

## Abstract

**Background:**

High-risk human papillomavirus (HR-HPV) is the primary cause of cervical cancer, leading to over 311 000 global deaths, mainly in low- and middle-income countries. Kenyan women living with HIV (WLHIV) face a disproportionate burden of HR-HPV.

**Objectives:**

We determined the prevalence of HR-HPV infections and their association with cervical cytology findings among Kenyan WLHIV.

**Method:**

We conducted a cross-sectional study among WLHIV attending the HIV care and treatment clinic at the Kenyatta National Hospital (KNH), Kenya’s national referral hospital. Study nurses collected a cervical sample with a cytobrush for HR-HPV genotyping using Gene Xpert^®^ assays and HPV Genotypes 14 Real-TM Quant V67-100FRT. Bivariate analysis explored the associations.

**Results:**

We enrolled 647 WLHIV (mean age of 42.8 years), with 97.2% on antiretroviral therapy (ART) and 79% with a suppressed viral load (< 50 copies/mL plasma). The prevalence of any and vaccine-preventable HR-HPV was 34.6% and 29.4%, respectively, with HPV 52 being the most common genotype (13.4%). Among WLHIV with HR-HPV infections, 21.4% had abnormal cervical cytology. Women with multiple HR-HPV infections were more likely to have abnormal cytology compared to those with single HR-HPV infections (34.9 vs 9.3%, adjusted odds ratio [aOR] = 6.2, 95% confidence interval [CI]: 2.7–14.1, *P* = 0.001). Women with HR-HPV infection (single or multiple) were more likely to be on the second-line ART regimen compared to those without HR-HPV infections (53.1% vs 46.7%, aOR = 2.3, 95% CI: 1.3–4.1, *P* = 0.005).

**Conclusion:**

Among WLHIV at KNH, abnormal cytology was common and more frequent among women with multiple HR-HPV infections.

**What this study adds**: These results add to the growing evidence that more efforts are needed to improve routine screening for high-risk human papillomavirus (HR-HPV) infections and cervical cancer among WLHIV as well as optimised HPV vaccination among eligible girls.

## Introduction

Cervical cancer is one of the AIDS-defining cancers and is ranked fourth among the most common cancers affecting women worldwide.^1,2^ In the year 2020, over 550 000 incidental cases of cervical cancer were reported, with a staggering 90% of these cases and 300 000 cervical cancer-related deaths occurring in low-income countries.^3^ Persistent infection with high-risk human papillomavirus (HR-HPV) types is responsible for more than 70% of global cervical cancer cases, with varying prevalence rates of HR-HPV infections observed among different countries.^4,5^ In a global context, eastern, western and southern African regions exhibit the highest incidence rates of cervical cancer, with age-standardised rates of 34.5, 33.7, and 26.8 cases per 100 000 populations, respectively. In Kenya, the annual incidence rate of cervical cancer is 15 cases per 100 000 women, and the corresponding annual mortality rate is 12 deaths per 100 000 women.^6^ This substantial disease burden can largely be attributed to insufficient access to screening services and suboptimal screening uptake rates, stemming from limited knowledge or apprehensions regarding cervical cancer and HR-HPV screening among women. Other factors that have led to a high HR-HPV disease burden include low vaccine coverage and high rates of HIV and HPV co-infection as well as limited access to screening services.^7,8^

The persistence of HR-HPV infections can result in the development of cervical lesions. Pre-cervical cancer lesions and cervical cancer lesions are most frequently observed in women aged 30 years and above,^9^ indicating that the infection occurs earlier in life and progresses slowly to cancer. Metaplastic changes in the endocervix continue to occur throughout a woman’s lifetime, with the period of greatest activity coinciding with the highest risk of HPV infection during puberty and the first pregnancy. Metaplastic changes gradually decline after menopause.^10,11^ Precancerous stages or pre-invasive precursor lesions are graded based on cytological examination and include atypical squamous cells of undetermined significance (ASCUS), low-grade squamous intraepithelial lesions (LSIL), high-grade squamous intraepithelial lesions (HSIL), and invasive cervical cancer (ICC).^12,13,14^

Women living with HIV are at a disproportionate risk of acquiring HR-HPV infections, harbouring multiple HR-HPV infections, and developing persistent infections that can result in abnormal cervical lesions due to their compromised immune status.^15,16^ In contrast, HR-HPV infections in HIV-negative women may be cleared in some individuals by the stable cell-mediated immune system or tend to regress.^17^ Although antiretroviral therapy (ART) has been associated with a decrease in the incidence of other AIDS-defining cancers, its impact on cervical lesions remains unclear. In HIV-infected women, cervical lesions and the progression to malignancy tend to occur at an earlier age (around 20–25 years) when there is immunosuppression and when other competing health factors are present. Due to the presence of multiple HR-HPV infections, invasive malignancies may progress more quickly to unusual locations and respond poorly to therapies. Additionally, the risk of recurrence from latent and sanctuary sites is high, resulting in a poor prognosis.^18^ The prevalence of HR-HPV infections among WLHIV on ART and resulting cervical cytology patterns remains unclear in low-income settings, with some studies reporting no effects of ART on cervical cancer.^19^

Primarily, cervical cancer is preventable through the vaccination of young girls aged 9–14 years with the HPV vaccine. The secondary prevention strategy for cervical cancer prevention is through cervical cancer screening among women aged 30 years and above, which has been implemented.^20,21,22^ However, annual reports indicate sustained cervical cancer incidental and mortality rates among WLHIV in the ART era in sub-Saharan Africa (SSA).^6^ The development of three different HPV vaccines that are strongly efficacious against some HR-HPV types offers protection against cervical infections caused by the HR-HPV genotypes in the vaccines as well as HPV-associated cancers and condylomas. The synergy between primary and secondary prevention efforts of cervical cancer among WLHIV is still unclear. One of the most neglected aspects of the potential effect of prophylactic vaccines in SSA is the evaluation of existing screening practices and existing HR-HPV genotypes. There may be unique challenges and considerations for this population regarding HPV vaccination and screening.^6^ Women living with HIV have a higher risk of developing HPV-related cancers due to their compromised immune systems, which may limit the effectiveness of the HPV vaccine.^17^ Furthermore, WLHIV may experience barriers to accessing both HPV vaccination and cervical cancer screening services due to stigma, discrimination, and limited healthcare resources.^23^ Therefore, it is crucial to consider these factors when implementing HPV vaccination and cervical cancer screening programmes for WLHIV.^24^

The World Health Organization (WHO) recommends novel approaches to increase the uptake and utilisation of cervical cancer screening and fast-track same-day ‘test and treat’ algorithms for WLHIV.^25^ The algorithm involves screening WLHIV for HR-HPV using a point-of-care HPV DNA molecular rapid platform, and, if the test is positive, performing visual inspection with acetic acid (VIA) and treating precancerous lesions with cryotherapy on the same day.^25^ HR-HPV DNA testing has been utilised as a screening and triage test due to its increased sensitivity.^26,27^ The algorithm can help to identify and treat precancerous lesions before they progress to cancer. The same-day treatment approach can also improve patient satisfaction and reduce the burden of multiple clinic visits. However, there are also potential barriers to implementing the ‘test and treat’ algorithm. One of the main challenges is the lack of trained healthcare providers and infrastructure in many low- and middle-income countries where the burden of HIV and cervical cancer is highest. The algorithm also requires reliable electricity, equipment, and supplies for HR-HPV DNA point-of-care consumables and cryotherapy, which may not be available in some settings.^28^ There is also a need for community engagement and education to increase awareness and uptake of cervical cancer screening among WLHIV.

There is limited information on the epidemiology of the current HR-HPV infections among WLHIV and the associated cervical cytology patterns in the ART era in SSA. With the increased risk of cervical abnormalities among WLHIV in Kenya and the further development of cervical cancer, there is an urgent need to invest more in interventions targeting HR-HPV genotype screening and prevention. These interventions largely require HR-HPV genotypes and cytological patterns data for effective implementation. Currently, there are no comprehensive insights into the prevalence rates and distribution of HR-HPV infections and their correlation with cervical cytology among Kenyan WLHIV enrolled for care and treatment.

## Materials and methods

### Study design and population

This cross-sectional study recruited WLHIV aged 18 years and above attending the ART clinic at Kenyatta National Hospital (KNH) from June 2021 to March 2022. Those who were willing to participate consented and were counselled on the benefits of cervical cancer screening. The study excluded the following participants: pregnant women, history of previous total hysterectomy, radiotherapy or chemotherapy, those currently menstruating, and those with abnormal vaginal bleeding.

### Study site and procedures

Kenyatta National Hospital is the largest tertiary health facility in Kenya, with a bed capacity of 1800 and a bed occupancy rate of 90% – 95%. The ART clinic at KNH was established 20 years ago for the prevention and treatment of HIV and associated morbidities in men and women including adolescents and children. On average, the ART clinic offers services to nearly 10 000 clients, with the highest proportion being women. Cervical cancer screening commenced 12 years ago as part of integrated service for HIV care for WLHIV at the ART clinic. Trained nurses performed routine annual visual inspections with acetic acid or Lugols iodine (VIA or VILI). Women found to be positive are offered cryotherapy or loop electrosurgical excision (LEEP), depending on the accessibility to the transformation zone, by the gynaecologists upon referral to gynaecological specialised clinic at KNH. Cytological screening and HPV genotyping of high-risk types are not routinely done for patients.

In this study, eligible WLHIV were offered initial partial HPV testing using Gene Xpert^®^ (Cepheid, Sunnyvale, California, United States [US]), and cytology smears were prepared for examination by a trained cyto-screener in the cytology laboratory at KNH. The additional individual HPV genotyping was done using HPV Genotypes 14 Real-TM Quant V67-100FRT (SACACE Biotechnologies^®^, Italy). Briefly, the study procedure involved face-to-face interviews after written informed consent was obtained. The interview covered sociodemographic, behavioural and sexual characteristics, history of HIV diagnosis, investigation, and treatment. Afterward, each participant was examined by a trained research nurse at the ART clinic, including a general physical and pelvic examination. In the lithotomy position, a nurse passed a sterile Cusco speculum to visualise the cervix, and a cytobrush was inserted into the endocervix and rotated 360 degrees twice. Part of the sample collected was used to prepare a slide for cytological assessment and the remaining samples were sent to the laboratory for HPV genotyping. The nurse also reviewed the HIV electronic medical records (EMR) of each participant and copied relevant information into the data collection form.

All participants had post-test counselling, irrespective of the HPV test and cervical cytology results. Women that had abnormal cytology using ASCUS classification (ASCUS: LSIL, HSIL, ICC) were referred to a gynaecologist for further evaluation and treatment based on KNH guidelines.

### Sampling procedure

All eligible participants at the ART clinic were informed as a group about the study before the commencement of the clinic session. Women that signified intention to participate in the study had further discussions with a research assistant individually on the study objectives and procedure. At each clinic session, a sampling frame was generated from a list of all eligible women in attendance. We used a simple random sampling technique to select eligible women from the sampling frame. In this technique, random numbers generated from Excel were used to select women before consent was taken.

### Biological sample storage and analysis

The slide for the cervical spear was prepared by the oncology-trained nurse using the standard protocol of KNH for Papanicolaou smear preparation. The prepared slides were shipped to the cytopathology laboratory for staining and reading by a cytopathologist. The results were interpreted using the Bethesda system.^14^ The leftover smear sample in the brush was placed in a PreservCyt solution (Hologic Corp, Marlborough, Massachusetts, US) and taken to the laboratory for HPV detection of high-risk HPV types (16, 18, 45, and 11 other HR-HPVs) using the Xpert HPV platform (Cepheid, Sunnyvale, California, US). Thereafter, advanced individual HR-HPV genotyping was conducted. First, the DNA was extracted using a QIAamp DNA Mini Kit in accordance with the manufacturer’s protocol (Qiagen, Crawley, United Kingdom [UK]). The HPV detection was done, and specific genotypes were then identified and interpreted using the HPV Genotypes 14 Real-TM Quant V67-100FRT kit (SACACE Biotechnologies^®^, Italy).

### Data collection and analysis

Data collected were entered into the ODK tool kit and exported as a CSV file before importing into SPSS^®^ version 23.0 (IBM Corp., Armonk, New York, US). Descriptive statistics were presented in frequencies and proportions for categorical variables and mean or median for continuous variables. The primary outcome of this study was the presence or absence of abnormal cervical cytology readings (LSIL, HSIL, and ICC). Bivariate analysis was also done to explore the association between sociodemographic, clinical characteristics, and lifestyle characteristics with having normal or abnormal cytology using Chi-square test of associations, after which a multivariate logistic regression model was built to adjust for potential confounders.

## Results

### Enrollment process

**FIGURE 1:**
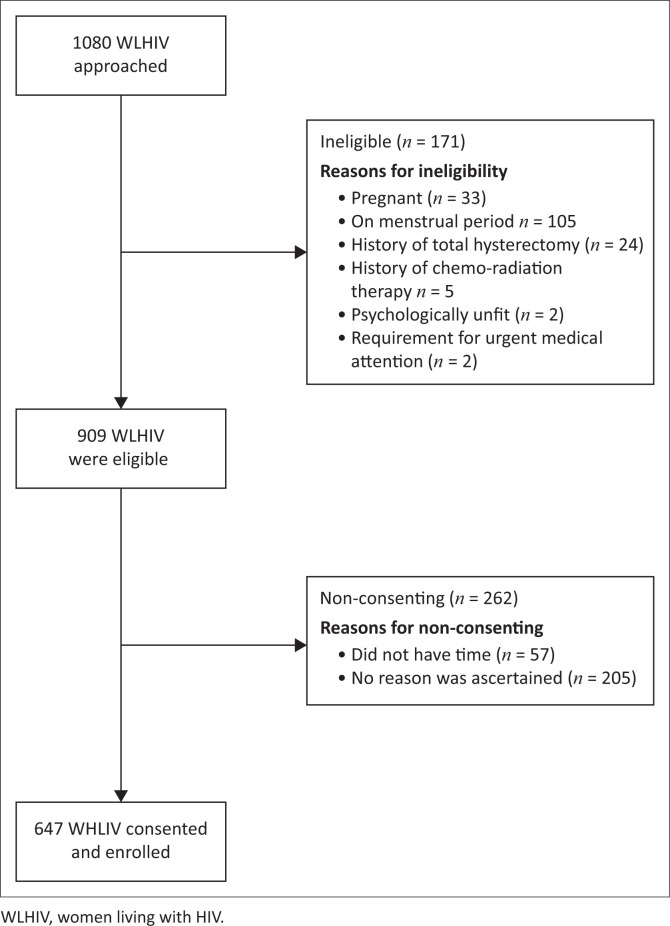
Flow diagram of the enrollment process.

As depicted in Figure 1, among the 1080 WLHIV initially approached, 171 were deemed ineligible and 262 of the approached women declined to participate, citing time constraints, while others refrained from providing their reasons for non-participation. Consequently, a total of 647 WLHIV provided their consent and actively engaged in the study.

### Social demographics, sexual and behaviour characteristics of the participants

Participant characteristics are shown in Table 1, the mean age of the participants was 42.8 years (standard deviation [s.d.]: 8.7) and the majority (40.5%) were aged from 35 years to 44 years. The mean age for sexual debut was 18.3 years (s.d.: 3.0) and 43% of the women had three or more children. Only 29.3% of the respondents were on a family planning method. Almost half of the respondents (43.4%) reported a history of sexually transmitted infection (STI) treatment in their sexual lifetime and 68.5% of the participants had a sexual partner currently or in the last 6 months. Most of the respondents (91.6%) had only one sexual partner and 46.7% of these sexual partners were HIV sero-reactive (HIV-concordant sexual partners). The majority of the participants (85.3%) reported that their sexual partners were circumcised. Only 1.1% of the participants were vaccinated against HPV.

**TABLE 1:** Social demographics, sexual and behaviour characteristics of the participants (*N* = 647).

Variable	Frequency (*n*)	%
**Age category (years)**		
< 25	15	2.3
25–34	91	14.1
35–44	262	40.5
45–54	215	33.2
55+	64	9.9
**Level of education**		
None	14	2.2
Primary	167	25.8
Secondary	263	40.6
College	203	31.4
**Employment status**		
Source of income	502	77.6
No source of income	145	22.4
**Family planning method**		
Condoms	61	9.4
Injectable	57	8.8
Intrauterine device	30	4.6
Tubal ligation	10	1.5
Oral contraceptive	4	0.6
Other contraceptives	37	5.7
Others	55	8.5
No method	393	60.7
**Number of children**		
None	50	7.7
1	131	20.2
2	188	29.1
3 or more	278	43.0
**Ever treated STIs**		
Yes	281	43.4
No	366	56.6
**Consistent condom use (male or female), *n* = 443**		
Yes	226	51.0
No	217	49.0
**Sexual partners**		
Yes	443	68.5
No	204	31.5
**Number of sexual partners, *n* = 443**		
1	406	91.6
2+	37	5.7
**Partner’s HIV status**		
Positive	207	46.7
Negative	152	34.3
Do not know	84	19.0
**Partner circumcised**		
Yes	378	85.3
No	62	14.0
N/A	3	0.5
**HPV vaccination**		
Yes	7	1.1
No	640	98.9
**Cigarette smoking**		
Yes	24	3.7
No	623	96.3
**Alcohol consumption**		
Yes	190	29.4
No	457	70.6
**Any other drug**		
Yes	12	1.9
No	635	98.1

Note: Age: Mean = 42.8; s.d. = 8.7; Age at sex debut: Mean = 18.3; s.d. = 3.0; Age at first pregnancy: Mean = 21.7; s.d. = 5.1; Age at HIV diagnosis: Median = 32.0; IQR = 26.0–38.0; Minimum – maximum = 1.0–63.0.

### An analysis of the clinical characteristics of the participants

As shown in Table 2, the majority of the participants (33.8%) were diagnosed with HIV when they were aged between 35 and 39 years. Viral load was suppressed (HIV-1 RNA viral load < 50 copies/mL plasma) in 79%, 12.3% had low-level viraemia (50 copies/mL – 999 copies/mL plasma) and 8.8% were unsuppressed (≥ 1000 copies/mL plasma). Approximately 37% of the participants had an absolute CD4 cell count of < 200 cells/µL. Almost all the participants (97.2%) were on ART, with 87.6% being on a dolutegravir-based regimen and 7.8% on second-line ART. Only 1.1% of the participants were HPV vaccinated. A few of the participants (20.9%) had missed their HIV clinical appointments and 25.5% had missed taking their daily ART at least once in the last 12 months.

**TABLE 2:** An analysis of the clinical characteristics of the participants (*N* = 647).

Variable	Frequency (*n*)	%
**Age (years) at HIV diagnosis**		
< 18	28	4.3
18–24	100	15.5
25–29	143	22.1
30–34	133	20.6
35–49	219	33.8
50–54	19	2.9
55+	5	0.8
**Current HIV RNA (*n* = 628)**		
Suppressed	496	79.0
Low-level viraemia	77	12.3
Unsuppressed	55	8.8
**CD4 WHO categorisation (*n* = 503)**		
< 200	184	36.6
200 to < 500	133	26.4
500+	186	37.0
**ART regimen**		
TDF+3TC+DTG	567	87.6
TDF+3TC+EFV	12	1.9
AZT+3TC+NVP	1	0.2
AZT+3TC+LPV/r	3	0.5
TDF+3TC+LPV/r	27	4.2
AZT+3TC+ATV/r	17	2.6
TDF+3TC+ATV/r	18	2.8
Any other regimen	1	0.2
**ART Regimen**		
1st regimen line	580	92.2
2nd regimen line	49	7.8
**HPV vaccination**		
Yes	7	1.1
No	640	98.9
**Missed ART clinic visit**		
Yes	135	20.9
No	512	79.1
**Missed daily ART intake**		
Yes	165	25.5
No	482	74.5

s.d., standard deviation; IQR, interquartile range; RNA, ribonucleic acid; CD4, cluster of differentiation 4; WHO, World Health Organization; ART, antiretroviral therapy; TDF, tenofovir disoproxil fumarate; 3TC, lamuvidine; DTG, dolutegravir; EFV, efavirenz; AZT, zidovidine; LPV/r, lopinavir/ritonavir; ATV/r, atazanavir/ritonavir.

### Overview of the overall prevalence of HR-HPV infections among WLHIV

**FIGURE 2:**
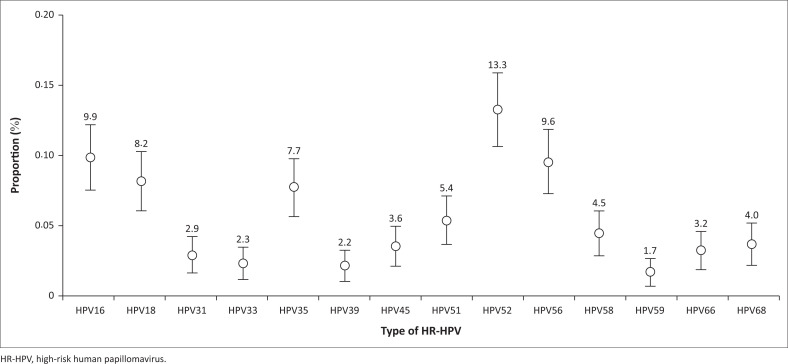
Overview of the overall prevalence of high-risk human papillomavirus infections among women living with HIV.

The prevalence of any and vaccine-preventable HR-HPV was 34.6% and 29.4%, respectively. As shown in Figure 2, the highest HR-HPV infection was type 52 (13.4%), followed by the types (in descending order): 16 (9.9%), 56 (9.6%), 18 (8.2%), 35 (7.7%), 51 (5.4%), 58 (4.5%), 68 (4.0%), 45 (3.6%), 66 (3.3%), 31 (2.9%), 33 (2.3%), 39 (2.2%), and 59 (1.7%).

### Prevalence of HR-HPV among WLHIV across different age categories

Based on age categories, WLHIV aged less than 25 years had the highest rate of HR-HPV infections (46.7%) followed by those aged 35–44 years (36.6%). Those aged 55 years and above had the lowest rate of infection (26.6%), as shown in the Appendix.

### Examining the pattern of abnormal cervical cytology among WLHIV having HR-HPV infections

**FIGURE 3:**
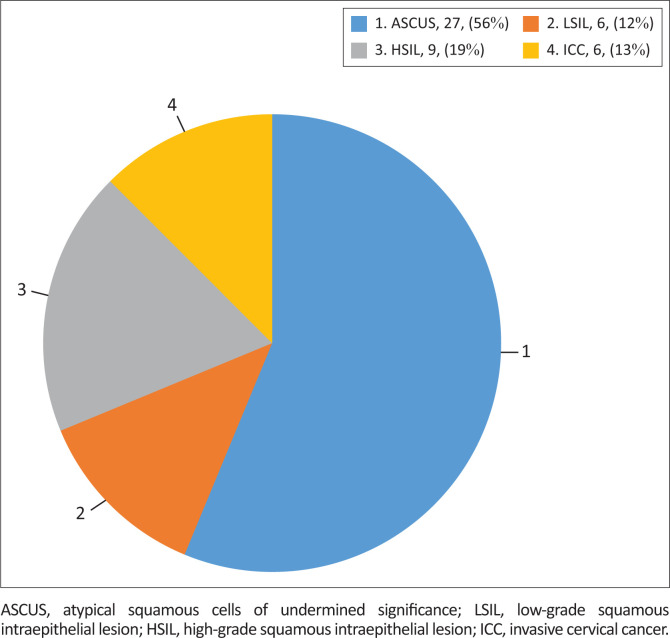
Pattern of abnormal cervical cytology among women living with HIV having high-risk human papillomavirus infections.

Out of the 224 WLHIV having HR-HPV infections, 21.4% had abnormal cervical cytology. Of those with abnormal cytology, 56% had ASCUS, 12% had LSIL, 19% had HSIL and 13% had ICC, as shown in Figure 3.

### The relationship between cervical cytology and HR-HPV infections among WLHIV

Among WLHIV with normal cervical cytology, the most dominant HR-HPV genotype was HPV 52 (30.7%). Participants having ASCUS had HR-HPV type 56 as the most detected genotype (81.5%), those with LSIL had HR-HPV 16 dominating (66.7%), and HR-HPV 31 dominated in HSIL (66.7%). Women living with HIV with cervical cancer had HR-HPV type 16 and 52 equally dominating (50% each), as shown in Table 3.

**TABLE 3:** The relationship between cervical cytology and high-risk human papillomavirus infections among women living with HIV.

HR-HPV types	Normal cervical cells		ASCUS		LSIL		HSIL		Cervical cancer	
	*n*	%	*n*	%	*n*	%	*n*	%	*n*	%
16	50	28.4	6	22.2	4	66.7	1	11.1	3	50.0
18	41	23.3	5	18.5	0	-	5	55.6	2	33.3
31	9	5.1	0	-	3	50.0	6	66.7	1	16.7
33	13	7.4	0	-	1	16.7	1	11.1	0	0
35	25	14.2	17	63.0	2	33.3	4	44.4	2	33.3
39	10	5.7	2	7.4	0	-	2	22.2	0	0
45	12	6.8	5	18.5	1	16.7	5	55.6	0	0
51	7	4.0	20	74.1	3	50.0	4	44.4	1	16.7
52	54	30.7	21	77.8	3	50.0	5	55.6	3	50.0
56	31	17.6	22	81.5	3	50.0	4	44.4	2	33.3
58	2	1.1	19	70.4	2	33.3	4	44.4	2	33.3
59	8	4.5	2	7.4	0	-	1	11.1	0	0
66	6	3.4	13	48.1	1	16.7	1	11.1	0	0
68	15	8.5	9	33.3	0	-	1	11.1	1	16.7

HR-HPV, high-risk human papillomavirus; ASCUS, atypical cells of undetermined significance; LSIL, low-grade squamous intraepithelial lesion; HSIL, high-grade squamous intraepithelial lesion; ICC, invasive cervical cancer.

### The relationship between cervical cytology (normal/abnormal) and selected characteristics

Participants aged 35 years and above were more likely to have abnormal cytology (24.7% vs 5.3%, adjusted odds ratio [aOR] = 7.27, 95% CI: 1.48–35.79, *P* = 0.015). Women living with HIV with multiple HR-HPV infections were more likely to have abnormal cytology as compared to those with single HR-HPV infections (34.9% vs 9.3%, aOR = 6.2, 95% CI: 2.7–14.1, *P* = 0.001). No other risk factor was significantly associated with abnormal cytology, as shown in Table 4.

**TABLE 4:** The relationship between cervical cytology (normal/ abnormal) and selected characteristics.

Selected characteristics	Abnormal		Normal		Univariate			Multivariate		
	Mean	s.d.	Mean	s.d.	OR	95% CI	*P*	OR	95% CI	*P*
**Age category**										
≤ 34	2	5.3	36	94.7	1.0	-	-	1.0	-	-
35+	46	24.7	140	75.3	5.91	1.37–25.53	0.017	7.27	1.48–35.79	0.015
**Employment status**										
Employed/salaried	40	22.9	135	77.1	0.7	0.4–1.5	0.408	0.8	0.3–2.4	0.731
Unemployed	14	28.6	35	71.4	1.0	-	-	1.0	-	-
**Age (years) at sexual debut**										
Categories	17.9	2.6	18.2	2.6	-	-	0.551	1.0	-	-
< 18	28	28.3	71	71.7	1.0	-	-	0.9	0.3–2.5	0.863
18–25	25	20.5	97	79.5	0.7	0.4–1.2	0.179	-	-	1.000
> 25	1	33.3	2	66.7	1.3	0.1–14.5	0.849	-	-	-
**Number of children**										
None	1	7.1	13	92.9	1.0	-	-	-	-	-
1	11	21.6	40	78.4	3.6	0.4–30.4	0.243	-	-	-
2	14	25.9	40	74.1	4.6	0.5–38.0	0.162	-	-	-
3 or more	28	26.7	77	73.3	4.7	0.6–37.8	0.143	-	-	-
**Duration of ART use in months**										
1st year	7	30.4	16	69.6	1.0	-	-	1.0	-	-
2nd–5th year	9	16.1	47	83.9	0.4	0.1–1.4	0.155	0.2	0.04–1.0	0.051
Above 5th year	32	22.1	113	77.9	0.6	0.2–1.7	0.380	0.3	0.1–1.3	0.112
**Ever treated STIs**										
Yes	27	23.9	86	76.1	1.3	0.7–2.6	0.364	-	-	-
No	21	18.9	90	81.1	1.0	-	-	-	-	-
**Sexual partners**										
Yes	33	21.0	124	79.0	0.9	0.5–1.8	0.819	0.7	0.3–1.7	0.454
No	15	22.4	52	77.6	1.0	-	-	1.0	-	-
**Number of sexual partners**										
None	18	26.9	49	73.1	1.0	-	0.343	1.0	-	-
1	28	20.9	106	79.1	0.7	0.4–1.4	0.471	-	-	-
2+	8	34.8	15	65.2	1.5	0.5–4.0	-	1.7	0.5–6.2	0.405
**Partner’s HIV status**										
Positive	9	20.0	36	80.0	1.0	-	-	-	-	
Negative	13	18.3	58	81.7	0.9	0.3–2.3	0.821	-	-	
Do not know	11	26.8	30	73.2	1.5	0.5–4.0	0.455	-	-	
**Consistent condom use (male or female)**										
Yes	11	16.7	55	83.3	0.5	0.2–1.2	0.112	0.6	0.2–1.5	0.247
No	25	27.5	66	72.5	1.0	-	-	1.0	-	
**Partner circumcised**										
Yes	28	22.0	99	78.0	0.8	0.3–1.9	0.588	0.4	0.1–1.3	0.139
No	8	26.7	22	73.3	1.0	-	-	1.0	-	-
**Partner ever treated for STIs**										
Yes	29	24.2	91	75.8	0.4	0.1–3.3	0.387	0.4	0.03–3.4	0.370
No	1	11.1	8	88.9	1.0	-	-	1.0	-	-
Don’t know	6	21.4	22	78.6	0.9	0.3–2.3	0.759	0.8	0.3–2.7	0.759
**HR-HPV**										
Multiple	37	34.9	69	65.1	5.2	2.5–10.9	< 0.001	6.2	2.7–14.1	0.001
Single	11	9.3	107	90.7	1.0	-	-	1.0	-	-
**Alcohol consumption**										
Yes	13	18.3	58	81.7	0.8	0.4–1.5	0.438	-	-	-
No	35	22.9	118	77.1	1.0	-	-	-	-	-
**Cigarette smoking**										
Yes	2	25.0	6	75.0	1.2	0.2–6.3	0.681	-	-	-
No	46	21.3	170	78.7	1.0	-	-	-	-	-

s.d., standard deviation; ART, antiretroviral therapy; STIs, sexually transmitted infections; HR-HPV, high-risk human papillomavirus; OR, odds ratio; CI, confidence interval.

### Association between HIV-related characteristics and HR-HPV infections among WLHIV

Women living with HIV having HR-HPV infection (single or multiple) were more likely to be on second-line ART regimen compared to those without HR-HPV infection (53.1% vs 46.7%, aOR = 2.3, 95% CI: 1.3–4.1, *P* = 0.005).

## Discussion

HPV is the most prevalent STI worldwide and is a well-known risk factor for the development of cervical cancer, especially in WLHIV.^29^ Antiretroviral therapy has been shown to reduce the incidence of other AIDS-defining cancers in WLHIV, but the prevalence of HR-HPV infections in this population is still a significant concern.^6^ In this current study, we aimed to investigate the prevalence of HR-HPV infections in WLHIV enrolled in ART care and their correlation with cervical cytology patterns. The prevalence of HR-HPV infections in our participants was 34.6%, which is lower than the prevalence reported in other studies conducted in SSA, which ranged from 54% to 81%.^16,30^ The difference in the prevalence of HR-HPV infections might be explained by the groups examined in earlier research that included WLHIV who had established cervical cancer.^16,31,32^ We enrolled participants without any cervical abnormalities, and the prevalence of HR-HPV infections was detected through screening. A high proportion (21.4%) of the participants with HR-HPV infections in our study were discovered to have different types of cervical abnormalities. This finding emphasises the importance of regular cervical cancer screening in WLHIV.

We found that HPV 52 was the most common HR-HPV genotype (13.4%), followed by types 16 (9.9%) and 18 (8.2%), while HPV 59 was the least prevalent (1.7%). Notably, types 16, 18 and 45 were not dominant in this study, a trend observed in other studies among WLHIV and the general population in the region.^33,34^ This finding is notable because these three HPV types are the most oncogenic and are responsible for the majority of cervical cancer cases worldwide. The lower prevalence of types 16, 18 and 45 in our study may be due to regional differences in HPV prevalence or differences in the population studied, differences in the sample size, and laboratory methods used for HR-HPV testing.^26^ Additionally, it is plausible that the prevalence of distinct HR-HPV genotypes exhibits temporal variations due to shifts in sexual practices or variances in HPV vaccination rates across diverse populations.^8^ The immune status of the WLHIV participants may have played a role in the different distribution of HR-HPV genotypes observed in this study. This highlights the need to focus on other HR-HPV infections in addition to types 16, 18, and 45 in WLHIV enrolled for ART care.

Previous studies in Kenya have also reported a high prevalence of HR-HPV 52 and 35.^34,35^ Cervical abnormalities from additional HR-HPVs have received little attention in low-income settings, indicating the importance of individual genotyping of all HR-HPV types and implementation point-of-care molecular screening in the same-day screen-and-treat strategy for cervical cancer screening among WLHIV. Screening for a limited subset of HR-HPV genotypes may not optimally identify those at risk of infection and cervical lesion development. According to the findings of this research, WLHIV face a heightened vulnerability to contracting multiple HR-HPV infections, such as HPV 52 and other HR-HPV genotypes that are not safeguarded by the existing Cervarix or quadrivalent Gardasil HPV vaccines offered in Kenya. Given these circumstances, there is a pressing requirement to initiate HPV vaccination using Gardasil-9, which has demonstrated comparable or superior efficacy against seven HR-HPV types.^36,37,38^

This study found that young WLHIV aged < 25 years had the highest prevalence of HR-HPV infection (46.7%). This increased prevalence among young women may be due to their heightened susceptibility to HR-HPV as a result of early sexual debut and increased sexual activity. Though not statistically significant in this study, participants who had their first sexual debut before 18 years of age were more likely to have HR-HPV infections as compared to others. Similar findings were reported by Guthrie et al. among WLHIV in discordant relationships in Kenya.^34^ While HR-HPV infections among young women may regress with time due to viral transience, persistence or latency may also occur, which could increase the risk of developing cervical cancer over time.^39^ These findings suggest the need for targeted interventions to address the high prevalence of HR-HPV infections among young WLHIV, particularly those aged less than 25 years. Such interventions should focus on promoting safe sexual practices, HPV vaccination, early detection and treatment of HR-HPV infections, and related conditions, such as abnormal cervical cytology. Therefore, ongoing monitoring and screening for HR-HPV infections and related conditions are critical for the long-term health of WLHIV, especially those in the younger age group. In addition, it was observed that among WLHIV aged 35 years and over, there is an increased prevalence of abnormal cytology in the presence of HR-HPV infections. The heightened occurrence of abnormal cytology can be attributed to the persistent and reactivated state of latent HR-HPV infections, which were acquired during earlier sexual activity. Therefore, it is inadequate to rely solely on HPV vaccination as an intervention to eliminate HR-HPV infections and cervical cancer. It is imperative to implement adequate routine cervical cancer screening, as emphasised by the Ministry of Health and Cubie and Campbell, to enable timely detection and management of cervical cancer in this vulnerable population.^40,41^

The immunocompromised state of WLHIV makes them susceptible to HR-HPV infections with increased persistence, leading to a higher incidence of cervical lesions compared to HIV-negative women. Kelly et al.,^15^ in their study among WLHIV in South Africa and Burkina Faso, demonstrated this vulnerability. Our study also found abnormal cytology in 21.4% of WLHIV with detectable HR-HPV infections, of which 68% were low-grade precancerous lesions and 32% were high-grade pre-cancer lesions. However, the prevalence of cervical lesions in our study was lower than that reported by Guthrie et al.^34^ in their study of WLHIV in discordant relationships in Kenya. Importantly, it is worth noting that spontaneous regression of cervical lesions is common in over 75% of women, and thus, these lesions should be viewed as reflective of an infection rather than a disease stage or progression. This implies that the interpretation of cervical lesions should be done carefully to prevent stigmatisation, anxiety, and fear among the screened women. Furthermore, clearance of HR-HPV infections should also be approached with caution since it may be misinterpreted as an inability of the screening method used to detect existing HPV infections, as Krings et al. emphasised.^42^

In the context of WLHIV, the co-occurrence of multiple HR-HPV infections is frequently observed. The findings of this study demonstrate a significant correlation between the presence of multiple HR-HPV infections and abnormal cervical cytology. This outcome is consistent with a prior investigation conducted in South Africa, which evaluated WLHIV with cervical intraepithelial neoplasia and found a similar association.^16^ Menon et al., in their study conducted in Western Kenya, demonstrated a 2.3-fold higher prevalence of cervical lesions in individuals with multiple HR-HPV infections compared to those with a single HR-HPV infection.^19^ This finding provides evidence to support the hypothesis that the presence of multiple HR-HPV infections increases the risk of developing pre-cervical cancer lesions if HR-HPV infections do not regress. The coexistence of multiple HR-HPV infections may lead to a higher HR-HPV viral load, increased persistence of the virus, and increased oncogenic potential, resulting in an increased likelihood of developing cervical lesions.^19^ Taken together, these results underscore the importance of vigilant monitoring for the presence of multiple HR-HPV infections among WLHIV, as they may serve as a predictor for the development of cervical lesions if they do not regress naturally or are managed promptly. The South African study further reported that participants with multiple HR-HPV infections were found to exhibit inadequate responses to therapy. This highlights the urgent need for increased investment in HR-HPV vaccinations, particularly among eligible girls, to prevent the occurrence of multiple infections. This recommendation aligns with the findings of a study conducted by Haque and colleagues, which reviewed insights and solutions for the elimination of HR-HPV infections and cervical cancer in SSA.^43^ The implementation of comprehensive vaccination programmes targeting multiple HR-HPV types has the potential to substantially reduce the burden of cervical cancer in this region. In the current study, only 1.1% of the participants had been vaccinated for HPV. This is similar to previous research conducted in Kenya on the uptake of the HPV vaccine, which reported poor uptake of the vaccine (33% for the first dose and 16% for the second dose). The poor uptake was associated with lack of awareness and education about the virus. In addition, their study also found that only 30% of parents had heard of the HPV vaccine, and only 13% had knowledge of its purpose. This lack of knowledge and awareness can lead to misconceptions about the vaccine and, ultimately, low uptake. To address this barrier, public education campaigns and community outreach programmes could be implemented to increase knowledge and awareness about the HPV vaccine.^44^ Cultural beliefs and practices have also been identified as barriers to HPV vaccine uptake in Kenya. Some parents believe that the HPV vaccine could encourage sexual promiscuity among their daughters, which goes against their cultural values. Additionally, some parents believe that the HPV vaccine is not necessary because their daughters are not sexually active.^45^ In addressing this barrier, it is important to engage with local communities and involve cultural leaders in public education campaigns. The cost of the vaccine has also been identified as a barrier to HPV vaccine uptake in Kenya for adolescent girls aged >14 years. Many parents do not have health insurance and are not able to access the vaccine through the national immunisation programme.^46^ It may be necessary to explore alternative financing mechanisms, such as public-private partnerships or donor funding, to increase access to the vaccine. Fear of side effects is another barrier to HPV vaccine uptake in Kenya. Some parents are concerned about the safety of the HPV vaccine and are worried about potential side effects, including the future fertility impacts on young girls. This fear can lead to low uptake of the vaccine, despite its proven effectiveness.^47^

Despite the decreasing incidence of other AIDS-defining cancers, HR-HPV infections and cervical lesions have not shown a similar decline among WLHIV who are using ART.^48^ Specifically, our study found that 22.1% of the participants had been on ART for more than five years and had abnormal cervical cytology results. These findings highlight the ongoing need for continued research and interventions to address the complex health needs of WLHIV, including the potential for co-infections and comorbidities such as HPV-related conditions. In the present study, there was also no significant association between unsuppressed viral load and HR-HPV infections. This denotes that there was no relationship between HR-HPV infections or abnormal cervical cytology, and poor HIV treatment outcomes indicated by the unsuppressed viral load. Contrary to our findings, other studies reported that women on ART for a shorter period and those with unsuppressed HIV viral loads were associated with increased HR-HPV infections and cervical abnormalities.^15^ In 2017, the WHO recommended immediate ART initiation, regardless of the WHO clinical stage or CD4 count.^49^ Thus, women on ART have an increased life expectancy due to less competing risk of dying from opportunistic infections. Due to the improved cell-mediated immune status leading to prolonged life, incidences of HR-HPV infections, persistence, and later development to cervical lesions could be on the rise.^50^

The acquisition of new HR-HPV infections among WLHIV may be linked to having multiple sexual partners, according to research conducted by Brown and Weaver.^51^ Although we found there was no statistical association between the number of sexual partners and HR-HPV infection rates in the overall population, 34.8% of the respondents had two or more sexual partners, which could have led to increased HR-HPV infections and abnormal cytology. Other studies have also reported a higher risk of HR-HPV infections among individuals with multiple sexual partners, such as Okunade et al. and Wencel-Wawrzeńczyk et al.^52,53^ The present study found that condom use was not significantly associated with either HR-HPV infections or abnormal cytology. However, lack of condom use in conjunction with multiple sexual partners have been linked to an increased risk of STIs such as HR-HPV infections with progression to abnormal cytology. A study conducted among women of reproductive age in Gambia reported an increased risk of HPV infections among participants with multiple sexual partners and those who did not use condoms.^39^ These findings suggest that interventions aimed at reducing the number of sexual partners and promoting condom use may be effective in reducing the risk of acquiring new HR-HPV infections among WLHIV.

Alcohol consumption and cigarette smoking have also been linked to new HR-HPV infections and persistence, as noted in research conducted by Soh et al.^54^ Smoking has been shown to impair the function of Langerhans cells, which act as immune cells in the cervical region, thus increasing the vulnerability of smoking women to HR-HPV infections. This was observed in studies conducted by Skinner et al.^55^ and Mayaud et al.^8^ In the present study, approximately 18.3% of the participants with abnormal cytology were using alcohol, and 25% smoked cigarettes. However, these lifestyle factors were not statistically significant. Tobacco-associated carcinogens have also been found in the genital tract of women and may act as co-factors for the development of cervical lesions, particularly in the presence of other coexisting factors such as HIV infection and alcohol consumption.^56,57^

Lifestyle-related factors, such as early initiation of sexual intercourse, cigarette smoking, and promiscuity, have been found to increase the risk of HR-HPV infections and progression to cervical lesions in alcoholic women.^58,59,60^ This suggests that interventions targeting these risk factors could be effective in reducing the incidence and persistence of HR-HPV infections and associated complications.

One of the strengths of this study is the inclusion of a relatively large sample of WLHIV with factors associated with HR-HPV infections and cervical cytology. However, it is important to acknowledge certain limitations of our study. Firstly, it was limited to WLHIV who are currently receiving ART and, therefore, the findings may not apply to women who are not on ART. According to Kenya Ministry of Health data, 96.6% of WLHIV who know their status in Kenya are currently on ART and therefore our sample is representative of most WLHIV in this setting. Additionally, since this was a facility-based study, the results may not be representative of the general population, where ART use may be less common. Without ascertaining any reason why, over 200 eligible women declined enrollment. The decliners were younger, aged 18–22 years. We were not able to send WLHIV with HR-HPV for histology or colposcopy, although participants with abnormal cytology were referred to the gynae-oncology clinic for further management and follow-up. Exploring the relationship between histology or colposcopy findings and HPV positivity among WLHIV would be an intriguing area to investigate further. Such research could provide insights into the diagnostic utility of combining these modalities and help optimise screening and management strategies for cervical abnormalities. We also suggest conducting future investigations to establish a correlation between multizonal HPV disease and WLHIV who initiate ART with a low CD4 count.

## Conclusion

This study found that more than one-third of WLHIV had HR-HPV infections. There was a strong correlation between the presence of abnormal cervical cytology and having multiple HR-HPV infections, regardless of ART duration, CD4 count and behavioural factors. It is noteworthy that HR-HPV genotypes 52 and 35 were found to have a higher prevalence, emphasising the importance of considering all HR-HPV genotypes in cervical cancer prevention, management, and elimination efforts. Furthermore, a majority of respondents with HR-HPV infections exhibited ASCUS. Overall, this study underscores the importance of ongoing monitoring and screening for HR-HPV infections and related conditions for the long-term health of WLHIV.
